# Cross-talk between TGF-β1 and IL-6 in human trabecular meshwork cells

**Published:** 2009-02-11

**Authors:** Paloma B. Liton, Guorong Li, Coralia Luna, Pedro Gonzalez, David L. Epstein

**Affiliations:** Department of Ophthalmology, Duke University, Durham, NC

## Abstract

**Purpose:**

To investigate the relationship between transforming growth factor beta-1 (TGF-β1) and interleukin-6 (IL-6) in human trabecular meshwork (HTM) cells as well as to identify the signaling pathway/s involved in the increased IL-6 expression that occurs in response to mechanical stress and TGF-β1.

**Methods:**

All experiments were performed in confluent monolayers of HTM cells at passage 3. Secreted IL–6 was quantified by ELISA. Levels of *IL-6* mRNA were evaluated by polymerase chain reaction (PCR) analysis. Activation of the *IL-6* and *TGF-β1* promoters was monitored by measuring  secreted alkaline phosphatase protein (SEAP) released into the culture medium by HTM cells infected with an adenovirus expressing the *SEAP* reporter gene that was controlled by either the *IL-6* promoter (AdIL6–SEAP) or the *TGF-β1* promoter (AdTGFβ1-SEAP). Cyclic mechanical stress (5% elongation, one cycle per second) was applied using the Flexcell System. Reagents used in this study included human TGF–β1, human IL-6, and the inhibitors for the p38 mitogen-activated protein kinase (MAPK; SB202190), c-jun NH_2_-terminal kinase (JNK; SP600125), extracellular-regulating kinase (ERK; PD98059), and TGF type I activin receptor (SB431542).

**Results:**

Incubation of HTM cells with TGF–β1 (5 ng/ml) resulted in a significant increase in the protein and mRNA levels of *IL-6*, which was significantly diminished in the presence of the inhibitors for p38 MAPK or JNK. No transcriptional activation of the exogenous *IL-6* promoter was observed following TGF-β1 treatment. In addition, the presence of inhibitors for the p38 MAPK, ERK, and TGF-β1 pathways significantly decreased the increased expression of IL-6 by cyclic mechanical stress. Furthermore, exposure of HTM cells to IL-6 (100 ng/ml) demonstrated the transcriptional activation of *TGF-β1* promoter, which was severely impaired by blocking the p38 MAPK pathway.

**Conclusions:**

Our results indicate that TGF-β1 participates in the regulation of basal expression and the stretch-induced expression of IL-6 and suggest the possible existence in cultured HTM cells of an autocrine loop between IL-6 and TGF-β1. We also found that p38 MAPK might play a contributing role in the maintenance of such a loop.

## Introduction

The conventional outflow pathway, composed of the trabecular meshwork (TM) and Schlemm's canal (SC), constitutes the main route by which the aqueous humor (AH) exits the anterior chamber of the eye, and it is the tissue primarily responsible for maintaining proper levels of intraocular pressure (IOP) [1]. The functional failure of the TM/SC outflow pathway is believed to cause the elevation of IOP commonly associated with primary open-angle glaucoma (POAG) [2,3]. It has been hypothesized that the TM/SC tissue may respond to transient changes in IOP by altering its AH outflow resistance [4-7], thus maintaining normal IOP levels. However, the molecular and physiologic mechanisms regulating such potential outflow pathway tissue homeostasis are far from being understood.

Our laboratory has long hypothesized that mechanical stress accompanying the elevated IOP-associated changes in outflow pathway morphology induces the release from TM cells of factors that might act in a homeostatic, regulatory manner to increase outflow facility and lower IOP by either altering the conductivity of SC or changing the geometry of the TM pathway for aqueous flow. Supporting this hypothesis, we have demonstrated that exposure of TM cells to cyclic mechanical stress induces the expression of transforming growth factor beta-1 (TGF-β1) and interleukin-6 (IL-6) in human trabecular meshwork (HTM) cell primary cultures as well as organ cultures of porcine anterior segments [8,9]. We additionally found that IL-6 increases outflow facility when administered to porcine perfused anterior segments. Furthermore, our studies revealed that TGF-β1 itself upregulated the expression of *IL-6* in HTM cells, suggesting that the initial activation of TGF-β1 may be one of the contributing factors leading to the induction of *IL-6* [8,9]. The molecular mechanisms participating in these inductions are unknown.

Mitogen-activated protein kinases (MAPKs) are a family of serine/threonine-specific protein kinases that respond to extracellular stimuli and regulate various cellular activities such as gene expression, mitosis, differentiation, and cell survival/apoptosis. Three MAPKs have been identified in mammalian cells, the extracellular-regulating kinase (ERK), the c-jun NH_2_-terminal kinase (JNK), and the p38 MAPK. MAPK signaling pathways have been implicated in the expression of cytokines in response to other stimuli [10,11].

Herein, we aimed at further exploring the relationship between TGF-β1 and IL-6 in HTM cells as well as at identifying the signaling pathway/s involved in the increased *IL-6* expression in response to mechanical stress and TGF-β1. The data presented in this study suggest the existence of an autocrine loop between TGF-β1 and IL-6 in cultured HTM cells and that p38 MAPK might play a pivotal role in the maintenance of such a loop.

## Methods

### Reagents

Recombinant human TGF-β1 and IL-6 proteins were purchased from Sigma (St. Louis, MO). SB202190 (p38 MAPK inhibitor), PD98959 (mitogen activated protein kinase kinase 1 [MEK1] inhibitor), SP600125 (JNK inhibitor), SB431542 (TGF type I activin receptor inhibitor), and actinomycin D were obtained from Sigma.

### Cell cultures

Primary cultures of human TM cells were prepared from cadaver eyes (ages 30–60) obtained less than 48 h post-mortem from donors with no history of eye disease as previously described [12] and were maintained at 37 °C in 5% CO_2_ in low glucose Dulbecco’s Modified Eagle Medium (DMEM) with L-glutamine and 110 mg/l sodium pyruvate supplemented with 10% fetal bovine serum (FBS), 100 μM non-essential amino acids, 100 units/ml penicillin, 100 μg/ml streptomycin sulfate, and 0.25 μg/ml amphotericin B. All the reagents were obtained from Invitrogen Corporation (Carlsbad, CA). All experiments were performed in the absence of serum. Cultures were serum-withdrawn for 16 h before the experiment.

### Measurement of IL-6 concentration

The levels of IL-6 protein released to the culture medium were quantified with a commercially available sandwich enzyme-linked immunoassay kit (Biosource International, Camarilla, CA) according to the manufacturer’s instructions.

### Cyclic mechanical stress application in cell culture

Primary cultures of HTM cells at passage 3 were plated on type I collagen-coated flexible silicone bottom plates (Flexcell, Hillsborough, NC). Once confluence was reached, culture medium was switched to serum-free DMEM, and cells were subjected to cyclic mechanical stress (5% stretching, one cycle per second) for the indicated times in each experiment, using the computer-controlled, vacuum-operated FX-3000 Flexercell Strain Unit (Flexcell, Hillsborough, NC). Control cells were cultured under the same conditions, but no mechanical force was applied.

### Quantitative real-time polymerase chain reaction (qPCR)

**Figure 1:**
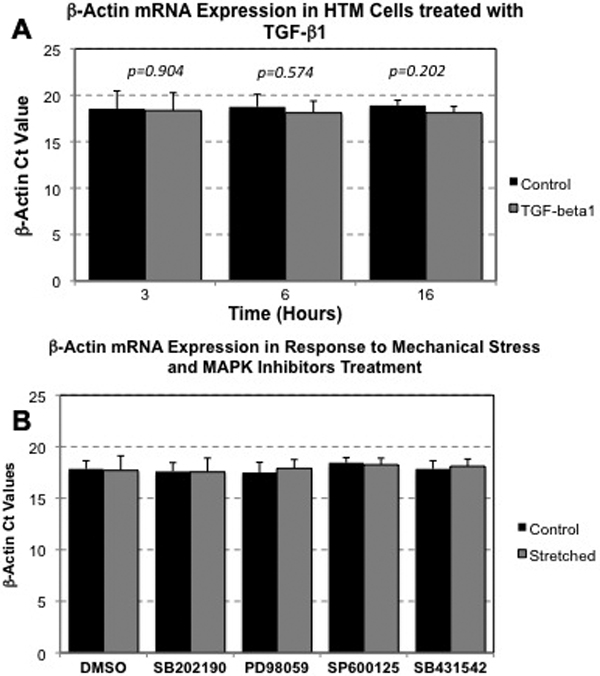
*β-Actin* mRNA expression. (**A**) *β-Actin* mRNA expression, expressed as a function of the C_t_ value, in HTM cells exposed to TGF-β1 (5 ng/ml) treatment during the indicated times. (**B**) *β-Actin* mRNA expression in HTM cells subjected to cyclic mechanical stress and MAPK inhibitors. Statistical significance between groups was assessed by the paired Student's *t*-test (n=3).

Total RNA from HTM primary cultures was isolated using RNeasy kit (Qiagen Inc., Valencia, CA) following the manufacturer’s protocol and then treated with DNase. RNA yields were determined using the RiboGreen® fluorescent dye (Molecular Probes Inc., Eugene, OR). First strand cDNA was synthesized from total RNA (1 µg) by reverse transcription using oligo(dT) primer and Superscript II reverse transcriptase (Invitrogen, Carlsbad, CA) according to the manufacturer’s instructions. Real-time polymerase chain reaction (PCR) reactions were performed in a 20 μl mixture containing 1 µl of the cDNA preparation, 1X iQ SYBR Green Supermix (Bio-Rad, Hercules, CA), and 500 nm of each primer in the Bio-Rad iCycler iQ system (Bio-Rad, Hercules, CA) using the following PCR parameters: 95 °C for 5 min followed by 50 cycles of 95 °C for 15 s, 65 °C for 15 s, and 72 °C for 15 s. The fluorescence threshold value (C_t_) was calculated using the iCycle iQ system software (Bio-Rad). Differential fold expression was calculated using the equation, 2^-ΔΔCt^, where ΔΔCt=ΔCt_experimental_−ΔCt_control condition_ and ΔCt=Ct_gene_–Ct_Act_. The absence of nonspecific products was confirmed by both the analysis of the melt curves and by electrophoresis in 3% Super AcrylAgarose (DNA Technologies, Gaithersburg, MD) gels. *β-Actin*, whose expression remained unchanged during all the experimental conditions (Figure 1), served as an internal standard for mRNA expression. The sequences of the primers used for the amplifications were: IL6 F 5′-CAA ATT CGG TAC ATC CTC GAC GGC-3′, IL6 R 5′ GGT TCA GGT TGT TTT CTG CCA GTG C-3′, β-Actin F 5′-CCT CGC CTT TGC CGA TCC G-3′, β-Actin R 5′-GCC GGA GCC GTT GTC GAC G-3′.

### SEAP reporter gene assay

HTM primary cultures were infected with 20 pfu/cell of either of the replication-deficient recombinant adenoviruses, AdIL6-SEAP [9] or AdTGFβ1-SEAP [8], which expresses the reporter gene, secreted alkaline phosphatase protein (*SEAP*), under the control of either the *IL-6* gene promoter (−1168/+3 region [13]) or the *TGF-β1* gene promoter (−453/+11 region [14], ), respectively. Activation of exogenous *IL-6* or *TGF-β1* promoters was quantified by determining the amount of the SEAP released to the culture medium through the use of the Great EscAPe^TM^ SEAP chemiluminescence detection kit (BD Biosciences Clontech, Palo Alto, CA) according to the manufacturer’s protocol.

### Statistical analysis

All the experiments were repeated in triplicate in independent experiments using three different HTM cell lines. Data are represented as mean±SD. Statistical significance between groups was assessed by the paired Student's *t*-test. A value of p<0.05 was considered statistically significant.

## Results

### Effect of TGF-β1 on *IL-6* expression in human trabecular meshwork primary cultures

To better characterize the TGF-β1 stimulatory effect on IL-6 secretion in HTM cells, we treated three different cultures of HTM cells with exogenous TGF-β1 in the absence of serum. The amount of TGF-β1 (5 ng/ml) used in these experiments was based on our previous study [9]. To not cause any additional stress, HTM cultures were allowed to equilibrate in serum-free media for 16 h before the addition of the cytokine.

**Figure 2:**
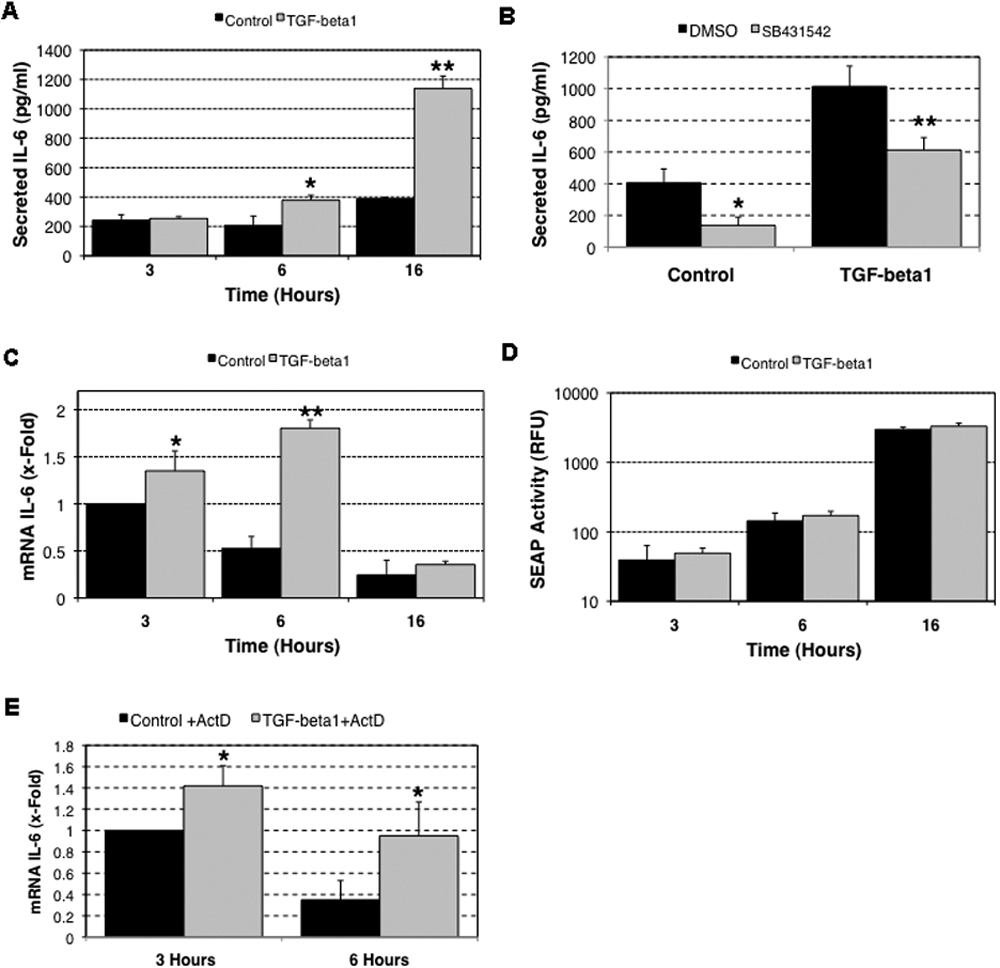
Effect of TGF-β1 on IL-6 expression in human trabecular meshwork primary cultures. Three independent primary cultures of HTM cells were serum-starved for 16 h and then incubated in serum-free media with TGF-β1 (5 ng/ml). **A**: Levels of secreted IL-6 quantified by ELISA are shown. **B**: Levels of secreted IL-6 in cultures pre-treated with the TGFRI inhibitor (SB431542) before TGF-β1 treatment is shown in the chart. **C**: Differential *IL-6* mRNA expression compared to the control at 3 h is shown. **D**: HTM cells were infected with AdIL6-SEAP (m.o.i=20 pfu/cell). At day 1 post-infection, cells were serum-starved for 24 h and then incubated in serum-free media with TGF-β1 (5 ng/ml). SEAP activity was assayed in the culture media at the indicated times. **E**: Differential *IL-6* mRNA expression of HTM cells that were exposed to TGF-β1 in the presence of actinomycin D (4 μg/ml) is shown. The asterisk indicates that p is less than 0.05, and a double asterisk denotes that p is equal to 0.001. Statistical significance between groups was assessed by the paired Student's *t*-test (n=3).

As shown in Figure 2A, exogenous TGF-β1 significantly produced an accumulation of secreted IL-6 in the culture media at 6 h (378.09±33.84 pg/ml IL-6 in treated cultures versus 206.7±62.01 pg/ml IL-6 in non-treated cultures, p=0.0137) and at 16 h (1,137.89±85.29 pg/ml versus 387.67±10.22 pg/ml, p=0.0001) post TGF-β1 treatment. The upregulated production of IL-6 in response to TGF-β1 treatment was markedly suppressed in the presence of SB431542, a TGF type I activin receptor inhibitor (Figure 2B; 1,014.12±128.36 pg/ml versus 613.2±78.1 pg/ml, p=0.004). Moreover, basal IL-6 secretion was also downregulated (Figure 2B; 405.63±87.1 pg/ml versus 137.9±52.3 pg/ml, p=0.01), indicating that endogenous TGF-β1 participates in the regulation of *IL-6* expression in HTM cells.

Quantification of *IL-6* mRNA by qPCR showed a progressive increase in *IL-6* mRNA concentrations following TGF-β1 treatment, which reached a peak at 6 h (1.80±0.088 fold, p=0.0001), and then declined to control levels after 16 h. It is noteworthy that *IL-6* mRNA levels decreased in the untreated cultures over time (Figure 2C).

To investigate whether elevated *IL-6* expression following TGF-β1 treatment is associated with increased *IL*-6 gene transcription, we examined the effect of TGF-β1 on *IL-6* promoter activity in HTM cells infected with AdIL6-SEAP. As shown in Figure 2D, TGF-β1 did not exert any significant effect on exogenous *IL-6* promoter activity in HTM cultures. Moreover, increased levels of *IL-6* mRNA following TGF-β1 treatment were observed in the presence of the transcription inhibitor, actinomycin D (4 μg/ml; Figure 2E).

### Effect of MAPK on the TGF-β1 induced expression of *IL-6*

**Figure 3:**
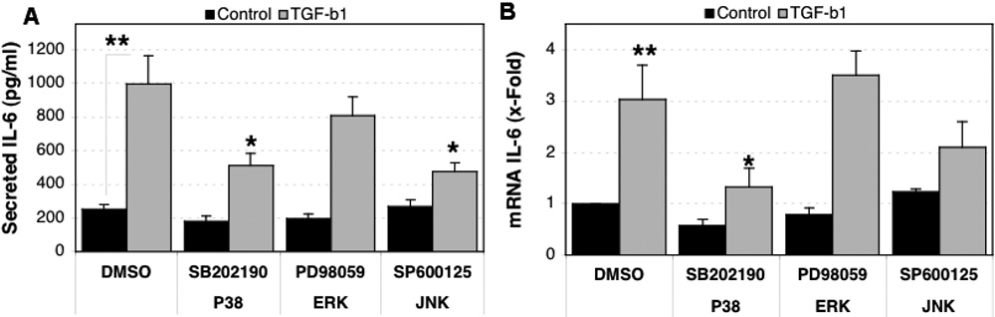
Effect of MAPK on the TGF-β1 induced expression of *IL-6*. Three independent primary cultures of HTM cells were serum-starved for 16 h, pre-treated for 2 h with the MAPK inhibitors, and then incubated in serum-free media with TGF-β1 (5 ng/ml) in the presence of the MAPK inhibitors (10 μM). **A**: Secreted IL-6 quantified by ELISA at 16 h post-treatment is shown. **B**: Differential *IL-6 *mRNA expression compared to control-DMSO, calculated at 6 h post-treatment. The asterisk indicates that p is greater than 0.001, and a double asterisk denotes that p is less than 0.001. Statistical significance between groups was assessed by the paired Student's *t*-test (n=3).

We next examined the intracellular signaling pathway through which TGF-β1 stimulates *IL-6* expression and secretion by HTM cells. For this, three different cultures of HTM cells that were serum-deprived for 16 h were pre-treated for 2 h with either p38 inhibitor (SB 202190, 10 μM), MEK1 inhibitor (PD98059, 10 μM), or JNK inhibitor (SP600125, 10 μM) and then exposed to TGF-β1 (5 ng/ml) in the presence of the inhibitors. As shown in Figure 3, pre-treatment of HTM with the p38 MAPK inhibitor led to a significant decrease in the TGF-β1 induced expression of *IL-6* at both the protein (513.43±72.53 pg/ml secreted IL-6 in SB202190-treated cultures versus 996.41±166.19 pg/ml in vehicle-treated cultures, p=0.009) and mRNA levels (1.31±0.37 fold *IL-6* mRNA expression in SB202190-treated cultures versus 3.03±0.67 fold in vehicle-treated cultures, p=0.017), quantified at 16 h and 6 h, respectively. Our results also showed a significant reduction in the levels of secreted IL-6 induced by TGF-β1 with the JNK inhibitor (474.41±55.68 pg/ml secreted IL-6 in SP600125-treated cultures versus 996.41±166.19 pg/ml in vehicle-treated cultures, p=0.007; Figure 3A). Although it did not reach statistical significance, *IL-6* mRNA was found to be downregulated with SP600125 (2.1±0.5 *IL-6* mRNA fold expression in SP600125-treated cultures versus 3.03±0.67 fold in vehicle-treated cultures, p=0.126). These data indicate a role of p38 MAPK and JNK pathways in *IL-6* induction by exogenous TGF-β1.

### Effect of TGF-β1 and MAPKs on the stretch-induced expression of *IL-6*

**Figure 4:**
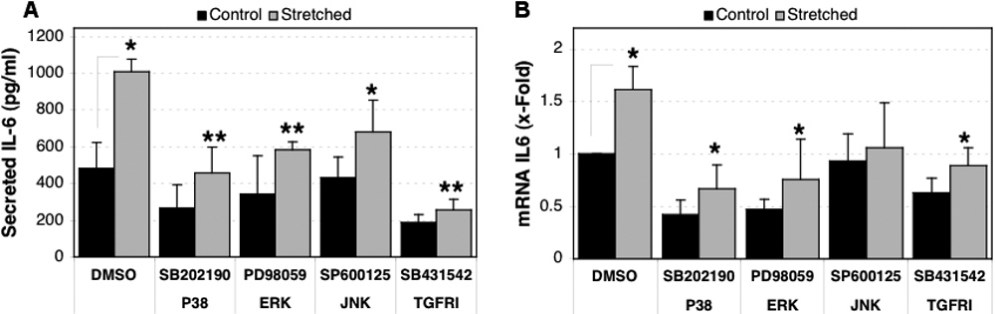
Effect of TGF-β1 and MAPKs on the stretch-induced expression of *IL-6*.Three independent primary cultures of HTM cells were serum-starved for 16 h, pre-treated for 2 h with the MAPK inhibitors (10 μM) or with the TGFRI inhibitor, and then subjected for 16 h to cyclic mechanical stress (5% elongation, one cycle per second) in the presence of the inhibitors (5 μM). **A**: Secreted IL-6 quantified by ELISA after 16 h of mechanical stress is shown in the chart. **B**: Differential *IL-6* mRNA expression compared to control-DMSO, calculated at 16 h of mechanical stress. The asterisk indicates that p is less than 0.05, and the double asterisk denotes that p is less than 0.005. Statistical significance between groups was assessed by the paired Student's *t*-test (n=3).

Our laboratory previously reported increased *IL-6* expression in HTM cells subjected to cyclic mechanical stress [8,9]. To characterize the signaling mechanisms by which mechanical stress upregulates *IL-6* in HTM cells, we pre-treated three different cultures of HTM cells, which were serum-starved for 16 h, with 10 μM of either SB202190 (p38 inhibitor), PD98059 (MEK1 inhibitor), or SP600125 (JNK inhibitor) for 2 h. Cultures were then subjected to cyclic mechanical stress (5% elongation, one cycle/second). As discussed elsewhere [8,9], this stretch regime was selected as an in vitro model to mimic the pulsatile mechanical forces to which TM are normally exposed [15,16]. Blocking the p38 or ERK pathway caused a significant decrease in the induction of *IL-6* by mechanical stress at both the protein (1,012.18±67.79 pg/ml with vehicle versus 458.6±142.74 pg/ml and 584.29±45.63 pg/ml with SB202190 and PD98059, respectively, p<0.005) and mRNA levels (1.61±0.22 fold with vehicle versus 0.66±0.22 fold and 0.75±0.38 fold with SB202190 and PD98059, respectively, p<0.05; Figure 4). Although some effect was also observed in the presence of SP600125, it varied among the different cell lines used in the study, suggesting that although it might participate somehow, JNK does not play a crucial role in the upregulation of *IL-6* mediated by mechanical stress.

We were also interested in investigating whether TGF-β1 might be involved in the stretch-induced expression of *IL-6*. For this purpose, we chemically blocked the TGF-β1 pathway using an inhibitor of the TGF type I activin receptor (SB431542, 10 μM). As shown in Figure 4, chemical inhibition of the TGF-β1 pathway clearly led to a significant decrease in the levels of both secreted IL-6 protein (1,012.18±67.79 pg/ml secreted IL-6 in stretched vehicle-treated cultures versus 256.27±57.6 pg/ml in stretched SB431542-treated cultures, p=0.0001) and mRNA content (1.61±0.22 *IL-6* mRNA fold expression in  stretched vehicle-treated cultures versus 0.89±0.17 fold in stretched SB431542-treated cultures, p=0.01) induced by cyclic mechanical stress. These data complement our previous observation showing diminished stretch-induced IL-6 production in the presence of neutralizing antibodies against TGF-β1 [9].

### Effect of IL-6 on the *TGF-β1* promoter activity

**Figure 5:**
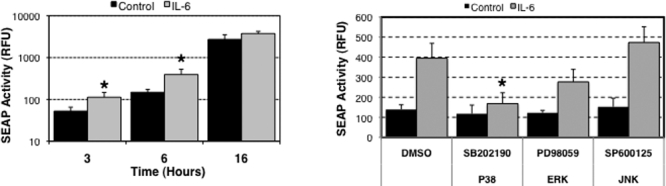
Effect of IL-6 on the *TGF-β1* promoter activity. Three independent primary cultures of HTM cells were infected with AdTGFβ1-SEAP (m.o.i=20 pfu/cell) and serum-starved for 16 h at day 1 post-infection. **A**: Cells were incubated in serum-free media with IL-6 (100 ng/ml). SEAP activity was assayed in the culture media at the indicated times. **B**: Cells were pre-treated for 2 h with the MAPK inhibitors and then incubated in serum-free media with IL-6 (100 ng/ml) in the presence of the inhibitors (10 μM). SEAP activity in the culture media was assayed at 6 h. The asterisk indicates that p is less than 0.05. Statistical significance between groups was assessed by the paired Student's *t*-test (n=3).

Several works have reported a cross talk between IL-6 and TGF-β1 signaling pathways in different cell lines [17-20]. We wondered whether IL-6 might upregulate *TGF-β1* expression in HTM cells and thus initiate an autocrine loop similar to the one described in activated pancreatic stellate cells [21]. For this purpose, we treated three different HTM primary cultures previously infected with the recombinant adenovirus, AdTGFβ1-SEAP, with IL-6 (100 ng/ml). Such IL-6 concentration, which significantly increased the permeability of SC endothelial cells as well as outflow facility in perfused porcine anterior segments [9], has been used for similar studies in other cell types [17]. As shown in Figure 5A, exogenous IL-6 quickly induced the transcriptional activation of *TGF-β1* promoter at 3 h (112.79±33.4 relative fluorescence units [RFU] in IL-6-treated cultures versus 52.34±12.79 RFU in control cultures, p=0.042) and 6 h post treatment (393.44±132.45 RFU  in IL-6-treated cultures versus 7.96±26.07 RFU in control cultures, p=0.034) compared to control levels. To identify the intracellular pathways participating in such activation, we repeated the experiment in the presence of MAPK inhibitors. Whereas PD98059 and SP600125 did not exert any major effect, SB202190 significantly decreased the percentage of induction in SEAP activity compared to the control (394.75±74.2 RFU in vehicle-treated cultures versus 168.43±53.8 RFU in SB202190-treated cultures, p=0.012), thus suggesting a role of p38 MAPK in the upregulation of *TGF-β1* by IL-6 (Figure 5B).

## Discussion

Our results indicate that TGF-β1 participates in the regulation of the basal and the stretch-induced expression of *IL-6* and suggest the possible existence in cultured HTM cells of an autocrine loop between IL-6 and TGF-β1. In addition, our data suggest that p38 MAPK might play a contributing role in the maintenance of such a loop. These findings support a potential role of mechanical stress in regulating AH outflow through a synergistic collaboration between MAPK and stretch-induced cytokines.

The upregulation of *IL-6* by TGF-β1 has been described in other cell types including human fibroblasts, osteoblasts, prostate cancer cells, and retinal pigmented epithelial cells [21-29]. Our time-course experiments showed a progressive accumulation of IL-6 in the culture media of HTM cells exposed to TGF-β1. Levels of *IL-6* mRNA were also found to be increased at early times (up to 6 h), returning to control levels after 16 h, thus indicating a transient effect of TGF-β1 on *IL-6* production. Strikingly, quantification of *IL-6* promoter activity using a recombinant adenovirus expressing the reporter gene, *SEAP*, under the *IL-6* promoter region showed no difference in *SEAP* activity between TGF-β1 treated and untreated HTM cultures at any time tested. However, a gradual increase in *SEAP* activity was detected over time in the untreated, control cultures, thus confirming the efficiency of the selected *IL-6* promoter region to mediate the expression of the reporter gene. Upregulation of *IL-6* is controlled by the activity of several transcription factors with known consensus sequences in the *IL-6* promoter region including IRF-1, AP-1, CRE, c/EBP, and NF-κB [30,31]. One might speculate that the promoter region included in our construct does not contain the binding sequence required for a particular transcription factor activated by TGF-β1. Although we cannot rule out the need of additional responsive elements in HTM cells, transactivation of the *IL-6* promoter by TGF-β1 has been reported to be mediated via an AP-1 responsive element (−283/−277) in human lung fibroblasts [22]. Such AP-1 binding site is contained within the promoter region used in our experiments. Furthermore, increased *IL-6* mRNA levels in response to TGF-β1 were also observed in the presence of the transcription inhibitor, actinomycin D. Thus, our results suggest that the high levels of expression of *IL-6* in response to TGF-β1 in HTM cells were not primarily mediated by a transcriptional activation of *IL-6*.

A potential mechanism by which TGF-β1 can mediate the observed increase in IL-6 is through mRNA stabilization. Many short-lived mRNAs encoding proto-oncogenes, nuclear transcription factors, and cytokines contain adenylate/uridylate (AU)-rich elements (AREs), which are usually found in the 3′-untranslated region and regulate mRNA stability. Stabilization of mRNA is a characteristic of short-lived cytokines and contributes to the strong and rapid induction of genes in the inflammatory response, which otherwise are targeted to degradation [32,33]. Indeed, *IL-6 *mRNA has been reported to rapidly be degraded in non-stimulated cells [18]. Similarly, our own results show a progressive decline in *IL-6* mRNA levels in untreated HTM cells. Induction of *IL-6* associated with mRNA stabilization has been described in several cellular systems in response to different compounds including TGF-β1 [17-20]. Additional, more specific experiments aimed at monitoring the half-life of *IL-6* mRNA will be required to confirm the stabilization of mRNA in HTM cells in response to TGF-β1 treatment.

MAPKs are known to be key regulators of the expression of many cytokines and in responding to extracellular stimuli. MAPKs are also known to play a crucial role in the response to cyclic stretching in cells from tissues that are subjected to this type of stress in physiologic conditions including the vascular endothelium, pulmonary epithelium, kidney podocytes, and cardiac myocytes [34-36]. Moreover, our previous studies, which used a static model of mechanical stress, have shown fluctuations in the levels of expression and phosphorylation of at least some MAPKs in HTM cells [37]. Therefore, we chose to investigate the potential participation of MAPKs in the stretch-induced expression of *IL-6*. We found that p38 MAPK and ERK1/2 pathways contribute to *IL-6* upregulation in stretched HTM cells and that their inhibition significantly attenuates *IL-6* protein and mRNA levels, indicating a synergistic collaboration of multiple signaling in the induction of *IL-6* by cyclic mechanical stress in HTM cells.

One of the most interesting observations in our study was that specific blockage of the TGF-β1 pathway using a TGF type I activin receptor inhibitor significantly affected the basal production of IL-6 and completely abrogated the increased expression of IL-6 with mechanical stress. These results clearly demonstrate a key role for TGF-β1 in basal IL-6 expression as well as the upregulated expression of IL-6 by cyclic mechanical stress in HTM cells. Since p38 MAPK was found to regulate both the TGF-β1 and the stretch-induced expression of IL-6, it is likely that the observed effect of TGF-β1 on the increased expression of IL-6 by mechanical stress is also partly mediated through p38 MAPK. Although TGF-β1 is known to regulate the expression of *IL-6* [21-29] and *IL-6* expression has been shown to be modulated by mechanical stress in different cell types [38-43], this is the first time to our knowledge that a connection between both events is established.

TGF-β1 has been reported to positively regulate its own transcription in TM cells [44]. Here, we observed that treatment of HTM cells with exogenous IL-6 induced the transcriptional activation of *TGF-β1* promoter and that such induction was impaired by blocking the p38 MAPK pathway. A similar autocrine loop between TGF-β1 and IL-6 was recently described in pancreatic stellate cells [21]. Altogether, these findings open up the possibility that activation of *TGF-β1* by mechanical forces might act as an upstream signal leading to increased *IL-6* expression, which in turn might upregulate the production of TGF-β1. We further propose that p38 MAPK might be one of the factors regulating such an autocrine loop between TGF-β1 and IL-6, although we do not discard the possibility of the synergistic collaboration of multiple MAPK pathways (in fact, our data suggest this possibility). Additional studies will be required to confirm the existence of a TGF-β1/IL-6 loop in HTM cells in response to mechanical stress.

In the short term, TGF-β1 and IL-6 potentially can have a beneficial effect in regulating the flow of AH. Indeed, we previously showed that exogenous administration of IL-6 produces an increase in outflow facility in porcine perfused anterior segments as well as an increase in permeability in cultured SC cells [9]. The existence of such a regulatory loop could offer important biological advantages by increasing redundancy in the mechanisms that modulate the expression of cytokines in TM cells and, perhaps more importantly, by providing a mechanism for amplification of relatively small stress signals. However, the permanent activation of these two pro-inflammatory cytokines can ultimately lead to undesirable secondary effects. Dysregulation of *IL-6* and *TGF-β1* has been linked to an array of inflammatory and pathologic conditions including atherosclerosis, Alzheimer disease, and rheumatoid arthritis [45-52]. Therefore, we anticipate the existence of a negative regulatory mechanism aimed at preventing a chronic activation of the stress response that might originate from the constitutive activation of such a loop under normal tissue physiology.

It is important to mention that a constitutive activation of the stress response controlled by an autocrine IL-1 feedback loop has been described in the cells within the glaucomatous outflow pathway [53]. In addition, a more recent study showed an altered responsiveness of ERK, p38 MAPK, and JNK pathways in glaucomatous TM cells, which the authors postulated might be due to the fact that the pathways are already maximally activated by the disease process [54]. In this scenario, TM cells could be unable to modulate their gene expression patterns and lose the flexibility to respond to the environment. Similarly, failure of the feedback mechanism controlling the TGF-β1/IL-6 loop could lead to a related disease phenotype.

In summary, we have described here that that TGF-β1 participates in the regulation of the basal and the stretch-induced expression of IL-6, and we suggest the possible existence of an autocrine loop between IL-6 and TGF-β1 in cultured HTM cells. We also found that p38 MAPK might play a contributing role in the maintenance of such a loop. Additional studies aimed at understanding the effects of mechanical stress on the MAPK pathways and their potential role in modulating the expression of TGF-β1 and IL-6 in HTM cells using mechanical stress models would very likely help to dissect the homeostatic molecular mechanisms involved in the outflow pathway “sensing” and “responding” to changes in IOP.
